# *In Vitro* Reporter Assays for Screening of Chemicals That Disrupt Androgen Signaling

**DOI:** 10.1155/2014/701752

**Published:** 2014-11-09

**Authors:** Gargi Bagchi Bhattacharjee, S. M. Paul Khurana

**Affiliations:** Amity Institute of Biotechnology, Amity University, Amity Education Valley, Panchgaon, Gurgaon, Manesar, Haryana 122413, India

## Abstract

Endocrine disruptive chemicals (EDCs) modulate hormone signaling and cause developmental and reproductive anomalies. Today, there is a global concern regarding endocrine disruption effects, particularly those mediated by the androgen receptor (AR). Androgen or male hormones are critical for the development and maintenance of male characteristics and numerous EDCs exist in the environment with the potential to disrupt androgen action. The threat is more during critical developmental windows when there is increased sensitivity to these compounds. Timely screening and detection of the EDCs is essential to minimize deleterious effects produced by these toxic chemicals. As a first line of screening,* in vitro* transcription assays are very useful due to their speed, convenience, and cost effectiveness. In this paper, recent* in vitro* reporter assays for detecting androgenic or antiandrogenic activity of EDCs have been reviewed. Two important cell systems used for this purpose, namely, the mammalian or yeast cell systems, have been discussed. Use of reporter genes such as bacterial luciferase (lux) and green fluorescent protein (gfp) has significantly improved speed and sensitivity of detection. Also, many of the current reporter assay systems can be used in a high throughput format allowing speedy evaluation of multiple potential EDCs at a lower price.

## 1. Introduction

EDCs are chemicals that interfere with the biosynthesis, metabolism, or action of endogenous hormones resulting in deviation from normal developmental programming and reproductive function [1]. The male sexual differentiation is entirely androgen-dependent and therefore highly susceptible to EDCs that disrupt androgen action. Androgens such as testosterone and its metabolite 5-*α*-dihydrotestosterone (DHT) exert their effect through the AR which is a ligand-induced transcription factor. Androgen binding causes the cytosolic AR to translocate into the nucleus, bind to the target regions of androgen-responsive genes, and influence their transcription [2]. Antiandrogens, on the other hand, may bind to the AR but do not promote nuclear translocation or gene transcription [3]. Recent findings indicate that an increasing number of natural products such as legumes, soybeans, flax, yams, and industrial chemicals, such as pesticides and fungicides, influence the activity of androgen and estrogen receptors [4]. These EDCs may have a profound impact on male reproductive health and androgen action including deterioration of sperm quality, increase in cryptoorchidism and hypospadias cases, alteration in sex ratio, and testicular dysgenesis syndrome [5]. Chemicals currently known to interfere with the androgen signalling pathway include dicarboximide fungicides such as vinclozolin, organochlorine-based insecticides such as p,p′-DDT and p,p′-DDE, conazole fungicides such as prochloraz, phthalates, and urea-based herbicides such as linuron [5]. The toxicity induced by these and other chemicals can be minimized only through thorough screening and early detection of their hazardous nature. The hazards of EDC accumulation were first revealed by studies which demonstrated changes in sex lives of fish in Duluth-Superior Harbor [6]. The chemicals in waste water accumulate in fish liver and also change egg production of female fish and mating behavior of male fish. Masculinized female mosquito fish was detected in rivers in the neighbourhood of pulp and paper mills [7]. Changes in characteristics of fish have been observed in both developed and developing countries of the world [8, 9].

To prevent EDC-induced toxicities, many countries have set out guidelines for the timely detection of such chemicals. A list of regulatory agencies from different countries involved in monitoring and controlling EDC exposure is provided in Table 1.

Typically, two levels of screens are conducted, a Tier 1 screening (T1S) to act as a “gate keeper” and a Tier 2 study (T2S) which is* in vivo* and more definitive. The first screen establishes whether a compound has potential for endocrine disruption and should be subjected to T2S. The T1S therefore emphasizes achieving maximum sensitivity, even at the cost of getting a few false positives [10]. The T1S includes (a) cell free receptor binding, (b) functional assays such as transcriptional activation or cell proliferation, (c) steroidogenesis using minced testes assay, and (d) additional enzyme assays.* In vivo* assays in rats, mice, or rabbits (used in T2S) were developed a long time ago to determine the endocrine activity of compounds. Important examples are the assessment of vaginal smear types to define estrogenicity [11] and of the prostate, seminal vesicle, and musculus levator ani (MLA) growth to determine androgenic and anabolic activities [12, 13]. The contribution of animal studies to EDC detection is restricted due to the costs involved, the desire to limit animal use, and speed.

Currently, the androgen levels are measured in clinical practice in mainly two ways. Immunoassays are based on the antibody's ability to recognize a specific chemical structure of the steroid molecule. These assays have variable specificity and sensitivity and the overall androgenic bioactivity in the sample cannot be correlated with the antibody binding detected because basis of antigen recognition is only structural and not functional. An alternative method of detection is gas chromatography-mass spectrometry (GC-MS). MS-based methods are powerful and very useful for use in sports doping laboratories as they are both specific and sensitive. Their major limitation is that they cannot identify compounds of unknown structure and rely on prior knowledge of structure of steroid. It is also a more labor intensive and expensive routine for everyday use [14].

## 2. The* In Vitro* Androgen Reporter Assays

The cell based reporter assays provide quantitative and functional information within a short span of time making them one of the most relevant and important assays for compound profiling and drug discovery. They measure the relative activity of a substance (or a mixture of substances) without the requirement of prior information about the chemical structure of the ligand.* In vitro* androgen receptor assays exploit the natural signaling pathway of androgens and compounds that bind to the AR. When ligands are added to the system, the receptor is activated and there is consequent production of reporter protein which can be measured. The routinely used reporter genes whose products can be assayed easily include luciferase, *β*-galactosidase, or green fluorescent protein. The commonly used reporter genes, their source, and functions are listed in Table 2.

The selection of an appropriate cell line is very important for the sensitivity and specificity of the reporter assay [15].

In this review we present an analysis of the currently used* in vitro* reporter assay systems that can be developed further as first line of screening (T1S) for detection of androgenic and antiandrogenic activity.

## 3. Mammalian Cells and Plasmids Used in Androgen Reporter Assays

**Figure 1:**
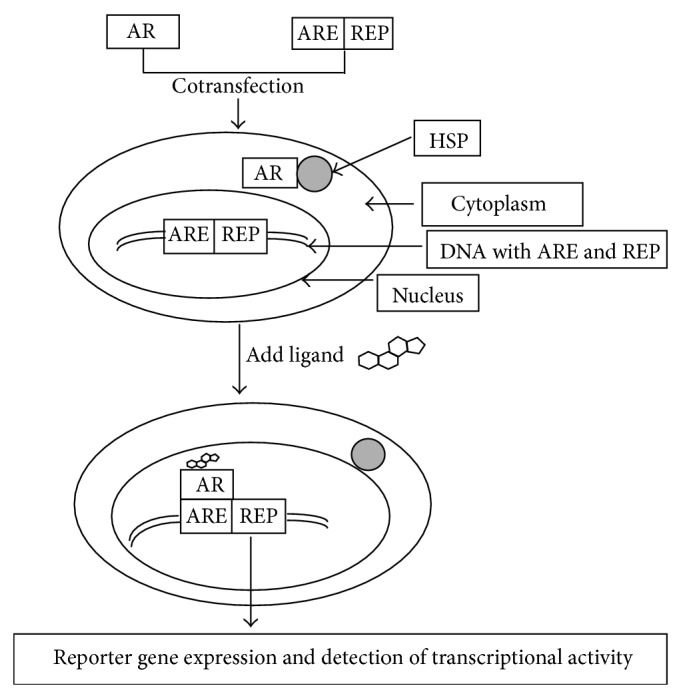
The principle of* in vitro* androgen transactivation assay, based on stable transfection of a cell line with two plasmids; one encoding the androgen receptor and the other, the androgen response element (ARE) upstream of a reporter (REP) gene such as luciferase. The unstimulated transfected cell expresses both AR and ARE-REP and the AR remains in cytoplasm bound to heat shock proteins (HSP). When the transfected cell is exposed to an androgen such as DHT, the AR moves into the nucleus, dimerizes, binds to ARE, and triggers expression of REP which can be monitored. The reporter gene expression correlates with bioactivity of androgen in the sample. Note: for simplicity only AR monomer binding to ARE has been depicted.

The cells used for developing* in vitro* AR transcription assays must fulfill two requirements: (a) express the AR and (b) carry a reporter system that allows measurement of androgen response. The reporter gene assays are based on the principle that when an androgen or antiandrogen enters the cell it binds to the AR in the cytoplasm; the androgen-AR complex enters the nucleus and binds to the androgen response element coupled to a reporter gene whose expression can be monitored (Figure 1).

Transient transfection assays in which both the AR and an AR-responsive reporter are cotransfected into native cells have been used for the purpose [16–18]. However, transient transfection assays may not reflect the endogenous level of receptors as the number of receptors may vary greatly in each system and from assay to assay. Moreover the response may be observed for a limited time since the transgenes are lost within 72 hours. Stably transfected cells can eliminate the need for repeated transfections and reduce variability. Because of their robustness and consistency they are being discussed in this review.

In a typical AR assay, a cell line such as CHO or MCF7 is transfected using a plasmid carrying the AR gene and another carrying a reporter gene such as luciferase, downstream of a sequence regulated by the AR. In stable transfection experiments, cells are checked for stable integration of both AR and reporter plasmids. Approximately 10,000 cells per well are plated on a 96-well plate, in 200 *µ*L of DMEM-F12 medium without phenol red and 10% FCS. The cells are washed with PBS on the following day and replaced by fresh medium. After about 3 h, the test compounds are added to the cells in a volume of 10 *µ*L in medium to achieve final concentrations of around 1 mM in ethanol. They are then further diluted in the medium in the final concentration of 0.01% before incubating them for another 24 h. Luciferase activity is measured using a kit. Compounds with androgenic activity show a luciferase activity that is significantly higher than that of control. For checking antiandrogenic activity, the test compound needs to be added to the stable integrated cells in the presence of an AR agonist such as R1881 or DHT. A significant decrease in R1881-induced luciferase reading in the presence of test compound indicates the presence of antiandrogenic activity.

Two types of cell lines have been routinely used for developing androgen transcription assays: mammalian cell lines and the laboratory yeast strain,* Saccharomyces cerevisiae*. The yeast cells have the advantage of rapid growth, low cost, and reproducibility. However, using yeast systems to express mammalian proteins can pose problems such as incorrect phosphorylation, glycosylation, folding, or other posttranslational modifications. Also, yeast systems lack the appropriate chaperone and coregulator proteins which are necessary for proper AR mediated transactivation.

When creating mammalian reporter cell lines, it is essential to express exogenous steroid receptors in cell lines with low background activity of other members of this receptor class. Unfortunately many of the cell lines traditionally used in the individual steroid receptor research do not fulfill this condition, and there is endogenous expression of more than one steroid receptor at a given time.

Many reporter plasmids and cell line specific assays have been developed in various laboratories within the last decade. Roy et al. [4] have developed a high throughput system to screen chemicals in a 96-well format. CHO-K1 cells were stably transfected using the AR and the luciferase reporter gene regulated by the hormone response element (HRE) present in the mouse mammary tumor virus (MMTV) promoter. The system has a high sensitivity (0.1 nmol/L) for androgens such as testosterone and can distinguish androgenic and antiandrogenic activities. However, low levels of endogenous glucocorticoid receptor (GR) expression in CHO-K1 cells can interfere with the androgen assay. The use of MMTV promoter may also add to the ambiguity of results as the HRE is responsive to both AR and GR. Being a high throughput system it is able to assess multiple samples at the same time.

Cell lines with endogenous expression of AR have also been used for AR reporter assay [19, 20]. The MDA-kb2 cell line developed from MDA-MB-453 breast cancer cells endogenously expresses AR and has been stably transfected with the MMTV-luciferase plasmid [19]. The expression level of AR in these cells was 240 fmol per mg protein and the lowest observed concentration that produced a response was 0.1 nM DHT. Although this is a sensitive test system, yet, expression of GR in the cells and use of MMTV HRE makes the assay less specific. Some compounds gave mixed response in this system, including hydroxyflutamide which acted as an antagonist at lower concentrations and agonist at higher concentrations.

Hartig et al. [20] used a monkey kidney cell line CV1 and the MDA-MB-453 breast cancer cell line, expressing endogenous AR, for developing reporter assays by adenovirus mediated transduction. The MDA-MB-453 based cells suffered from the same problems encountered with the MDA-kb2 cells as they expressed the GR. The CV1 cells, however, showed forty-five-fold activation in the presence of 0.1 nM DHT and the interference due to other receptors was minimal.

By fusing multiple copies of a hormone response element to a minimal promoter containing only the TATA box, Sonneveld et al. [21] have developed a series of highly sensitive and specific reporter cell lines called the CALUX (Chemically Activated Luciferase eXpression) cell lines. They stably transfected a human bone cell line U2-OS using the AR and the HRE associated minimal promoter linked to luciferase gene. The EC_50_ of DHT was 0.13 ± 0.02 nM using this assay. The cells did not show significant response to androgen precursors or GR ligands, though a response was induced upon exposure to high concentration (0.1 *µ*M) of dexamethasone. This was one of the most sensitive and specific reporter assays developed in a mammalian cell line. Xu et al. developed an androgen reporter system using African monkey kidney cell line CV-1 [22, 23]. Their use of CAT reporter was an improvement over the original *β*-galactosidase reporter system and resulted in an EC_50_ of 0.39 nM for DHT. This was however a transient reporter assay system and therefore prone to variations in different batches of transfections. A comparative analysis of advantages and disadvantages of all the mammalian reporter systems discussed above has been presented in Table 3.

## 4. Yeast Based Reporter Systems

Yeast cells have the advantages of fast growth, easy handling, cheap media components, and robustness towards toxic effects of test chemicals or solvents. Also, in yeast, activity of substances towards AR can be determined without the presence of any other mammalian proteins influencing the AR pathway. These factors together make yeast AR screen a fast and easy tool. Many different groups have therefore used the yeast reporter system for screening AR agonists and antagonists. Many yeast based detection systems involve either the colorimetric detection [24, 29] or the firefly luciferase reporter [29]. Chatterjee et al. [26] have developed a yeast based reporter system using human AR and ARE driven *β*-galactosidase. Production of* lacZ* is driven by the CYC1 yeast promoter. EC_50_ was 16 nM for testosterone and 4 nM for dihydrotestosterone. The use of* lacZ* reporter in this assay system required long exposure times for development of signal [30].

Recently the* Photorhabdus luminescens* lux operon has been substituted for the* lacZ* gene in yeast androgen reporter screen (YAS) assay (*S. cerevisiae* BLYAS), [27]. BLYAS strain contains human AR gene incorporated into its chromosome. Androgen-responsive elements were present in plasmids that also contained constitutively expressed luxA and luxB genes. Sanseverino et al. [28] have screened potential hormonally active chemicals using the BLYAS assay. This offers greater sensitivity (1.1 ± 0.5 × 10^−8^ for dihydrotestosterone) as compared to the original* lacZ* reporter. One significant advantage of bioluminescence assay is speed. Quantifiable bioluminescence is observed in 60 seconds with maximum signal detection in 3-4 hours. BLYAS can be used in high throughput. Exogenous reagents are not necessary for reporter signal development which reduces costs and manipulations. Interassay variability of BLYAS is also less. An obstacle in the use of BLYAS assays is chemical solubility as chemicals insoluble in methanol could not be evaluated. Adding hydrophobic chemicals directly to yeast medium may increase its usefulness; however, nonspecific solvent effects on bioluminescence and potential yeast toxicity need to be monitored. Another reporter system which has recently come to use is the green fluorescent protein, GFP [24, 25]. The reporter GFP emits green light that can be measured directly from culture without disintegrating the cells. Also chromophore formation can occur without any other cofactor. In the AR transactivation assay by Beck et al. [25], using GFP reporter, the source of AR was human AR from pSVARO plasmid. Androgen response element containing a consensus AR binding sequence composed of two 6 bp asymmetrical elements separated by a 3 bp spacer was used. The authors performed the assay using both *β*-galactosidase and the GFP reporter. The potencies obtained with *β*-galactosidase and GFP in the presence of testosterone were 27 nM and 23 nM, respectively, and 16 nM for both in case of dihydrotestosterone. The high backgrounds of GFP fluorescence made detection of GFP signals slightly difficult in their system. A comparative analysis of all the yeast based AR reporter assay systems has been provided in Table 3.

## 5. Discussion and Conclusions

Numerous reports and human studies have provided evidence for the existence of EDCs of natural and industrial origin that can specifically alter androgen signaling [1]. These may affect normal male developmental programming by interfering with androgen biosynthesis, metabolism, or action. Identification of these compounds by rapid, robust, inexpensive, and sensitive screening tests is essential for minimizing handling and exposure to these chemicals. In this review the* in vitro* reporter assay systems based on androgen receptor transcription have been analyzed and their advantages and disadvantages have been highlighted.

Androgen reporter assays can easily detect EDCs that alter androgen signaling by mimicking androgens or by blocking the classical androgen receptor transcription pathway. Sensitivity of the reporter systems is important and the first level screening emphasizes sensitivity more than anything else. Raivio et al. [31] have used a novel method for increasing sensitivity by introducing the AR-interacting protein 3 coactivator. Other AR coactivators such as p160, p300, and/or CARM1 [32] could also be incorporated into reporter systems for increasing the sensitivity of AR reporter assays. Sensitivity and ease of screening are also boosted by using different reporters such as GFP and Lux [27, 28, 30] instead of the traditional *β*-galactosidase or luciferase based systems.

The mammalian and yeast cell lines have both been used extensively for screening. Although yeast systems are inexpensive and robust, yet yeast does not contain all the mammalian enzymes, activator, and coregulators and hence may not support transcription of all compounds that would otherwise influence transcription in a mammalian system. Also, the yeast based assays are typically less sensitive than the mammalian cell based assays. Mammalian cell lines contain most of the coregulators and are better in this regard. However since some coregulators are tissue or cell specific, cell lines from androgen-responsive tissues such as prostate, testes, and fibroblasts may be more appropriate for these studies. Kim et al. [15] have used three different prostate cancer cell lines, in a transient androgen transfection assay. In their studies PC3/AR^+^ cells showed a 14-fold response in the presence of 10^−12^ M DHT and are one of the most sensitive androgen reporter assays.

The commonly used enhancer region used in the mammalian cell line based assays is the MMTV-LTR promoter [4, 15, 19]. A major disadvantage of this enhancer system is its response to glucocorticoids and progesterone apart from androgens. A natural androgen-responsive transcriptional enhancer from the rat probasin gene regulatory region has also been used [21, 26]. However, the HRE within this enhancer too can be recognized by glucocorticoid and progesterone receptors. Thus there is a need to identify and use an enhancer that is specific to androgen receptors in the mammalian systems. In this regard, the yeast cells represent a great advantage compared with mammalian cell lines as there is a lack of known endogenous receptors in yeast.

Sonneveld et al. have made comparisons of relative agonistic activities of 34 chemical compounds by* in vitro* reporter assays versus* in vivo* Hershberger assay in orchidectomized male rats [33]. The correlation of AR CALUX data with Hershberger assay resulted in a correlation coefficient of *r* ^2^ = 0.46 (*P* < 0.0001) indicating that correlation was not strong. However, the response of individual compounds* in vitro* was almost always able to predict the outcome* in vivo*, in their studies. This discrepancy between the two studies could be due to the fact that the physiological concentration of androgen required to elicit a response in the Hershberger assay is high (160 *µ*g/kg of testosterone) and therefore weaker androgens do not reach the activating concentration. Their study highlights that though cell based reporter assays are overall good indicators of agonist or antagonistic activity* in vivo*, yet considering the complex physiology of whole animals, more studies need to be performed to verify the correlation between* in vitro* reporter assay systems and* in vivo* animal test systems.

## Figures and Tables

**Table 1:** List of countries and regulatory agencies that have devised rules for monitoring EDCs.

S. number	Country	Regulatory agency	Mandate/strategy
1	USA	EPA-EDSP	Two-tiered *in vitro* and *in vivo *assays to identify and classify substances relative to their potential interaction with endocrine systems (Tier 1) and then to develop concentration-response relationship in animal models (Tier 2).

2	Japan	Environmental Agency-SPEED	(1) Promotion of field investigations into the present state of environmental pollution and of adverse effects on wildlife of endocrine disrupting chemicals. (2) Promotion of research and screening and testing method development. (3) Promotion of environmental risk assessment, risk management, and information dissemination. (4) Strengthening of international networks.

3	European Union	European Commission	(1) Short term strategy: to establish a priority list of candidate substances for further evaluation of their ED properties. (2) Medium-term strategy: the European Commission has made funding of research linked to ED a priority for their fifth, sixth, and seventh Framework Programmes. (3) Long term strategy includes development and adaptation of legislative instruments and policy action that enable hazard identification, risk assessment, and risk management of EDCs.

**Table 2:** Commonly used reporter genes and their characteristics.

S. number	Reporter gene	Function	Advantages	Disadvantages
1	*β*-Galactosidase (*lacZ) *	First reported in 1980. In *E. coli*, hydrolyses lactose to glucose and galactose.	Can act on many substrates.	Costly and potentially toxic chemical for assay and lysis of cells. Not useful for real time detection systems.

2	Luciferase (eukaryotic or bacterial)	Proteins that generate luminescence biologically. Can be eukaryotic or bacterial (lux). Firefly luciferase is one of the most common reporter genes.	High sensitivity, tight coupling of Luc protein with luminescence output, protein requires no posttranslational modification.	Firefly luciferase requires addition of costly substrate luciferin to monitor activity. The substrate for bacterial luciferase is produced endogenously, but not very active in eukaryotic systems.

3	GFP	Originally isolated from Jelly fish *Aequorea victoria*; gene was cloned later.	Functional in both prokaryotic and eukaryotic systems. Broad host applicability in absence of cell lysis or substrate addition.	These stable proteins continue to emit fluorescence long after the host has died. The fluorophore within wild type GFP needs two hours for generation.

4	CAT	The enzyme is found in prokaryotes. Transfers acetyl group from acetyl coA molecule to chloramphenicol, causing its detoxification.	Gene product is stable and detectable at attomolar concentrations. Suitable for mammalian systems.	Not suitable for high throughput studies.

**Table 3:** Comparative analysis of *in vitro* reporter systems in mammalian and yeast cells.

Cell line	AR source	Reporter plasmid	Reporter gene	Advantage	Disadvantage	Min^*^ dose	Reference
CHO-K1	hAR	MMTV-Neo-luc	Luciferase	Distinguishes androgen/antiandrogen activities	Expresses low levels of endogenous GR	0.1 nM	Roy et al., 2004 [4]
MDA-kb2	hAR	MMTV-Neo-luc	Luciferase	Endogenous expression of AR	Expresses GR.	0.1 nM	Wilson et al., 2002 [19]
MDA-MB-453	hAR	MMTV-Neo-luc	Luciferase	Endogenous expression of AR.	Expresses GR	0.1 nM	Hartig et al., 2002 [20]
U2-OS	hAR	3x HRE- TATA-luc	Luciferase	Highly specific assay system	AR activation by Dex and progesterone	0.13 nM (EC_50_)	Sonneveld et al., 2005 [21]
CV1	hAR	MMTV-CAT	CAT	Rapid, high fold activation	Transient transfection	0.39 nM (EC_50_)	Xu et al., 2008. [23]
Yeast	hAR	p406-ARE2-CYC1-yEGFP	GFP	Robust, minimum cross talk	Low sensitivity	33 nM	Bovee et al., 2007 [24], Beck et al., 2008 [25]
Yeast	hAR	ARE-*β*gal	*la* *cZ*	Sensitive	Long exposure time	4 nM	Chatterjee et al., 2007 [26]
Yeast	hAR	pUTK 404	Lux	Immediate luminescence detection	High background	9.7 nM	Eldridge et al., 2007 [27]
Yeast	hAR	pUTK 404	Lux	Immediate luminescence detection	Solubility of test compounds	5 nM for DHT	Sanseverino et al., 2009 [28]

^*^indicates the minimum dose which evokes a significant response.
